# Machine Learning Classification of Physiological Dynamics During Standardized Task-Demand Transitions

**DOI:** 10.3390/bioengineering13080898

**Published:** 2026-08-08

**Authors:** Elena Kriklenko, Anastasia Kovaleva

**Affiliations:** Federal Research Center for Innovator and Emerging Biomedical and Pharmaceutical Technologies, 125315 Moscow, Russia; info@academpharm.ru

**Keywords:** cognitive workload assessment, heart rate variability (HRV), support vector machine (SVM), neuroergonomics, adaptive system, operator performance

## Abstract

A major barrier to developing adaptive human–machine systems is the lack of interpretable physiological markers for characterizing physiological responses associated with changes in task demands. The aim of this study was to evaluate the feasibility of machine-learning classification of physiological responses during simple and complex task stages and two standardized task-demand transitions—baseline/rest-to-simple and simple-to-complex—in cognitive and motor-cognitive protocols. Sixty-nine healthy volunteers completed cognitive tasks involving normal and 180° inverted-text reading and motor-cognitive tasks involving simple and complex movement sequences performed with the non-dominant hand. Each protocol comprised a 1 min baseline followed by three consecutive blocks, each including a 1 min simple task, a 1 min complex task, and a 1 min rest period. Photoplethysmography (PPG), skin conductance (SC), and abdominal respiration were recorded continuously. Pulse-to-pulse (PP) intervals were extracted from the PPG signal, and conventional heart rate variability (HRV) indices, including mean RR interval, HR range, SDNN, RMSSD, Total Power, and SD2, were calculated in Kubios HRV from the PPG-derived interval series. Logistic Regression, Random Forest, and Support Vector Machine models were trained using either absolute physiological values or dynamic features (Δ%) calculated for the baseline/rest-to-simple and simple-to-complex transitions. Models based on dynamic features consistently demonstrated higher classification performance than those based on absolute physiological values. For the motor-cognitive protocol, Random Forest achieved an accuracy of 0.726 and a ROC-AUC of 0.730, whereas for the cognitive protocol, Support Vector Machine achieved an accuracy of 0.655 and a ROC-AUC of 0.722 on the held-out participant-level test set. The most informative features included mean RR interval (derived from PPG), Total Power, RMSSD, SDNN, SD2, and HR range in both protocols. Comparable feature-importance patterns across protocols suggest overlap in the physiological variables contributing to classification under the standardized experimental conditions. These findings are limited to the fixed transition sequence used in the present study and require confirmation in randomized or counterbalanced designs.

## 1. Introduction

The objective assessment of task complexity and human functional state during task performance remains a major challenge in neuroergonomics and biomedical engineering, as modern work environments increasingly require continuous monitoring of physiological state to prevent overload, optimize human performance, and reduce the risk of human-factor-related errors [1]. In recent years, growing attention has been devoted to the identification of physiological measures capable of assessing human functional state directly during task performance [2,3,4]. Such approaches are particularly relevant for operator performance monitoring, personnel training, adaptive human–machine systems, and digital learning environments, where timely detection of increasing task demands may improve both safety and efficiency. Recent advances in physiological sensing technologies enable continuous physiological monitoring of human state and create new opportunities for the development of intelligent physiological computing systems capable of adapting to the current condition of the user [5]. Consequently, there has been growing interest in the automatic classification of task complexity based on physiological measurements.

Recent advances in neuroergonomics have accelerated the integration of machine learning with multimodal physiological monitoring for continuous assessment of human functional state. Current research is increasingly focused not only on estimating workload but also on developing adaptive systems capable of responding to ongoing physiological changes in real time [6]. Nevertheless, recent reviews emphasize substantial methodological heterogeneity in feature extraction, model evaluation, and reporting practices, which complicates direct comparison between studies and limits the reproducibility and generalizability of published models. Traditionally, workload and task complexity have been assessed using subjective rating scales, such as the NASA Task Load Index (NASA-TLX), performance-based metrics, and expert evaluations [7]. Although these approaches provide valuable information, they have several important limitations. Subjective rating scales are typically retrospective and may interfere with ongoing task performance. Furthermore, they are inherently influenced by individual perception and interpretation. Performance-based measures often become available only after task completion and provide limited information about the physiological resources expended during task execution. For applications requiring continuous assessment, such as operator training and performance monitoring, physiological markers are particularly valuable because they can be recorded continuously and non-invasively during task execution [5,6,7,8].

Among peripheral physiological signals, heart rate variability (HRV), electrodermal activity, photoplethysmography (PPG), and respiratory parameters are the most used measures for assessing workload and functional state. HRV reflects autonomic regulation of the cardiovascular system and provides time-domain, frequency-domain, and nonlinear metrics associated with physiological flexibility, sympathovagal balance, and cardiac vagal control [9,10,11]. Electrodermal activity is highly sensitive to sympathetic nervous system activation and is widely used as an indicator of mental effort, arousal, and task-related activation [12,13]. Photoplethysmographic and respiratory measures provide complementary information regarding autonomic and vascular responses to changing workload conditions [14]. More recent studies have demonstrated that PPG-derived cardiovascular features are sensitive to changes in cognitive workload and provide informative biomarkers for physiological workload assessment during mental tasks [15]. Systematic reviews consistently demonstrate that no single physiological parameter is sufficient for accurately characterizing workload-related changes, whereas multimodal combinations of physiological signals generally provide better discrimination of human functional state [8,16,17]. This observation is particularly relevant for task complexity classification because increasing task demands rarely manifest as isolated changes in a single physiological variable. Instead, they are typically accompanied by coordinated reconfiguration across cardiovascular, electrodermal, respiratory, and peripheral vascular systems. To identify such complex physiological patterns, machine learning approaches have become increasingly important for the assessment of workload, stress, fatigue, and cognitive state [4]. Early neuroergonomics studies demonstrated that psychophysiological measures could successfully classify operator functional state and estimate workload in real time [18]. Subsequent research expanded this concept by integrating multimodal neurophysiological and physiological signals into supervised machine learning frameworks, including Support Vector Machines, Random Forests, artificial neural networks, and other classification algorithms [6,19,20]. Machine learning methods are particularly attractive because they capture nonlinear relationships between physiological variables and task demands that cannot easily be identified using conventional statistical approaches. At the same time, biomedical applications require not only high predictive performance but also physiological interpretability of model outputs. Consequently, classical machine learning algorithms such as Random Forests and Support Vector Machines remain highly relevant, particularly for datasets of moderate size where feature-importance analysis and physiological interpretation are essential [21].

Recent studies further demonstrate that multimodal physiological monitoring combined with machine learning enables accurate estimation of cognitive workload across a variety of experimental conditions [22]. In particular, Pontiggia et al. (2026) [23] emphasized the importance of developing robust multimodal classifiers capable of maintaining reliable performance across different physiological conditions and recording scenarios. Nevertheless, the primary emphasis of these investigations has been on maximizing classification performance, whereas comparatively less attention has been paid to identifying generalizable physiological mechanisms underlying adaptation to increasing task complexity.

Despite substantial progress in physiological workload classification, several important limitations remain. First, most recent studies have evaluated physiological responses within a single experimental paradigm, including driving simulation, aviation, vigilance, office work, or laboratory cognitive tasks, without examining whether the identified physiological signatures generalize across fundamentally different forms of human activity [22,24]. Furthermore, systematic reviews emphasize considerable heterogeneity in experimental protocols, physiological features, and machine learning methodologies, making direct comparison between studies difficult and limiting the identification of common physiological markers of task complexity [6,8].

Second, a large proportion of existing ML models rely on static physiological features calculated over predefined task intervals, whereas transient physiological responses occurring during transitions between consecutive task stages remain largely unexplored. Such transition periods are of particular interest because physiological regulation is inherently dynamic, reflecting continuous autonomic adaptation rather than stable physiological states. Third, the literature often considers workload as a unitary construct, although increasing task complexity may involve not only cognitive demands but also motor coordination, action switching, executive control, and sensorimotor integration. Finally, although recent ML models frequently achieve high classification accuracy, many rely on complex feature representations or black-box approaches that provide limited insight into the physiological mechanisms underlying model decisions. Consequently, there remains a need for physiologically interpretable models capable of identifying whether similar adaptive physiological features contribute across different forms of human activity [6,25,26].

One promising approach to addressing these limitations is the use of dynamic physiological features, which characterize changes in physiological regulation during transitions between consecutive task conditions rather than describing isolated physiological states. Absolute physiological measurements are strongly influenced by interindividual variability, including baseline autonomic activity, age, sex, physical fitness, emotional state, and other subject-specific factors. In contrast, relative physiological changes may directly reflect autonomic adaptation to changing task demands by reducing the influence of baseline differences between individuals [25]. Consequently, dynamic physiological representations may characterize regulatory responses associated with predefined changes in task demand. However, they should not automatically be interpreted as context-independent markers of complexity when the preceding condition differs between classes.

From a physiological perspective, transitions between simple and complex task conditions are expected to evoke rapid autonomic reconfiguration involving coordinated cardiovascular, electrodermal, respiratory, and peripheral vascular responses. These adaptive processes may not be adequately represented by average physiological values calculated over individual task periods. From a machine-learning perspective, transition-based physiological features may provide more robust descriptors of task-related adaptation by emphasizing relative physiological responses while reducing the influence of interindividual baseline variability [10]. Such features may therefore improve not only classification performance but also the physiological interpretability of ML models.

To our knowledge, few studies have directly compared conventional absolute physiological measurements with transition-based features calculated for repeatedly reproduced task-demand transitions across cognitive and motor-cognitive activities using a unified protocol and identical machine learning framework. Although recent studies have improved the robustness of multimodal physiological workload classification [23], the comparative value of absolute versus transition-based physiological representations across different activity modalities remains largely unexplored. Furthermore, previous investigations have primarily addressed physiological workload within individual task domains, whereas the existence of common physiological markers of increasing task complexity across different activity modalities remains largely unexplored. Addressing this question is essential for developing generalized physiological classifiers that extend beyond a single experimental paradigm and provide physiologically interpretable assessment of human functional state.

The present study aimed to evaluate the feasibility of machine-learning-based classification of task complexity using physiological signals recorded during cognitive and motor-cognitive activities. Specifically, we compared models trained on absolute physiological values recorded during simple and complex task stages with models trained on dynamic physiological features calculated for baseline/rest-to-simple and simple-to-complex transitions. The fixed sequence was selected deliberately to obtain three comparable observations of each transition type for every participant. The study was therefore designed to assess classification within this standardized sequence rather than to establish a context-independent classifier of task complexity. Unlike previous studies, the proposed framework enables direct comparison of physiological adaptation across two distinct activity modalities while preserving model interpretability through physiologically meaningful features and Random Forest feature-importance analysis.

We hypothesized that (1) dynamic physiological features would provide better discrimination between the two standardized transition types than absolute physiological values would provide between the corresponding task stages; (2) nonlinear machine learning algorithms would outperform linear classification models owing to the nonlinear nature of physiological adaptation; and (3) similar physiological feature patterns would be observed across both cognitive and motor-cognitive tasks, suggesting partial overlap in the physiological variables contributing to classification. By addressing these questions, the present study contributes to the development of physiologically interpretable and generalizable machine learning approaches for objective assessment of task complexity across different forms of human activity.

## 2. Materials and Methods

### 2.1. Participants

The study included 69 healthy volunteers aged 18–35 years (mean age: 23.69 ± 4.9 years): 34 females and 35 males. All participants had normal or corrected-to-normal vision and reported no history of neurological, psychiatric, or cardiovascular disorders.

All participants provided written informed consent prior to participation. The study was conducted in accordance with the Declaration of Helsinki and approved by the local ethics committee.

No formal a priori power analysis was performed because the sample size was determined by the number of participants available within the predefined experimental protocol. The final sample size is comparable to those reported in previous physiological workload studies.

### 2.2. Experimental Design

This study included two experimental protocols designed to create varying levels of cognitive and motor-cognitive complexity during continuous task performance.

All experimental sessions were conducted in standardized laboratory conditions during daylight hours, under controlled ambient temperature and lighting. Physiological signals were continuously recorded throughout each session.

**Figure 1:**
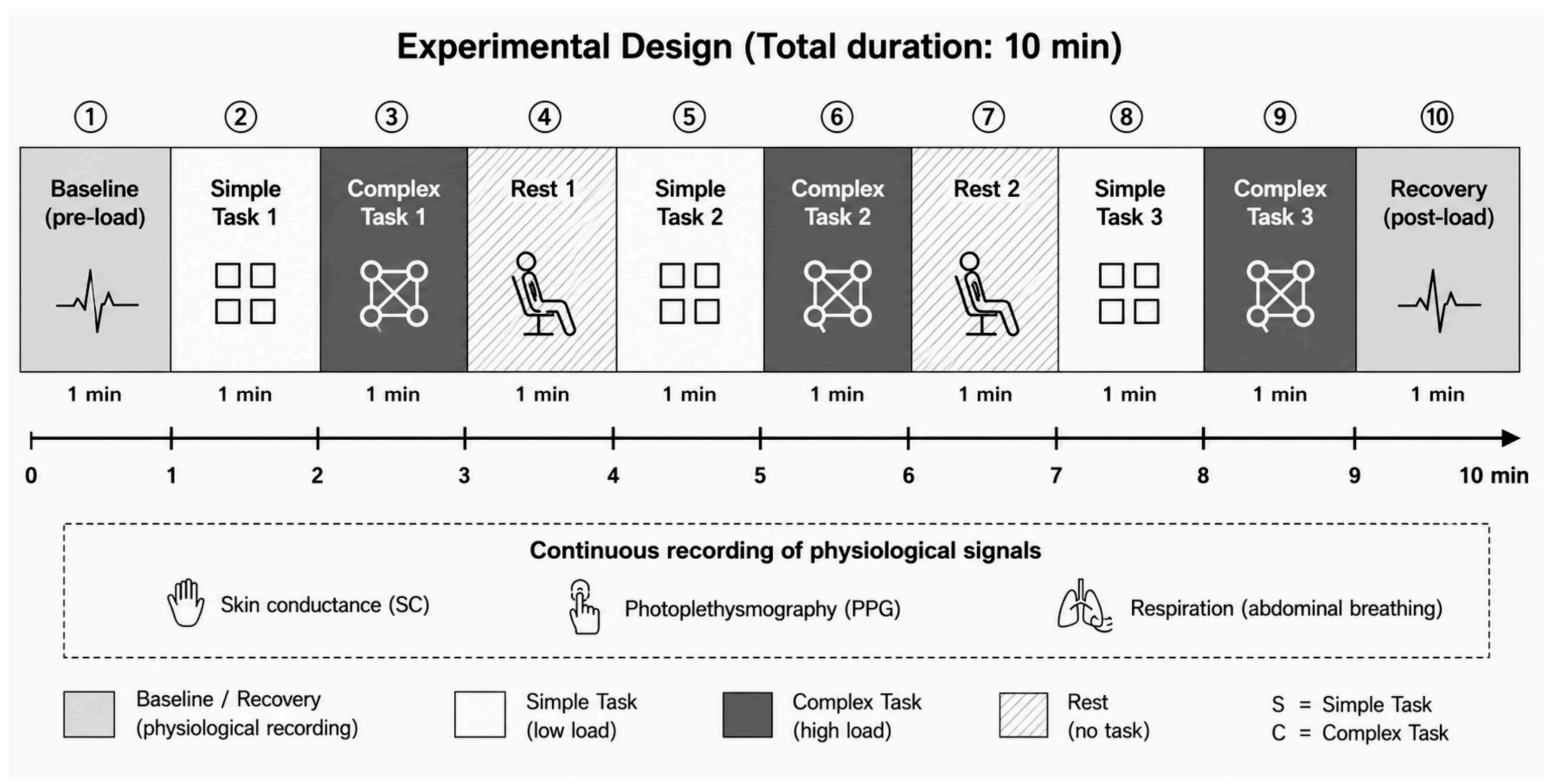
Experimental protocol. Physiological signals were continuously recorded during a 10 min session consisting of a 1 min baseline period, three repetitions of simple and complex task conditions separated by rest periods, and a 1 min post-task recovery period. The stage order was fixed and repeated three times to obtain comparable observations of baseline/rest-to-simple and simple-to-complex transitions.

Segmentation of the task into simple and complex stages was synchronized using software markers inserted during data collection (Figure 1).

The order of experimental stages was fixed by design. The principal purpose of this sequence was to obtain three repeated observations of two predefined increases in task demand: baseline/rest-to-simple and simple-to-complex. Randomization of individual stages would have generated additional transition types and substantially reduced the number of comparable observations per transition. Obtaining an equivalent number of observations for all possible transition combinations would also have required a longer protocol, potentially increasing fatigue and time-on-task effects.

#### 2.2.1. Cognitive (“Reading”) Protocol

The cognitive protocol consisted of alternating blocks of simple and complex reading tasks. Each task block lasted 1 min and was separated by 1 min rest intervals.

The simple condition involved reading standard horizontally oriented text, whereas the complex condition involved reading text rotated by 180°. Text stimuli were presented on a 15-inch monitor using automated slide presentation software.

The protocol comprised repeated cycles of (1) simple reading, (2) inverted-text reading, and (3) rest. The sequence was repeated three times. Before the beginning of the task series, a baseline resting recording was obtained.

The inverted-text condition was used to increase cognitive demand by introducing additional visuospatial transformation and perceptual processing requirements while preserving linguistic content.

#### 2.2.2. Motor-Cognitive (“Movement”) Protocol

The motor-cognitive protocol included tasks with different levels of motor and cognitive complexity.

The simple condition consisted of the sequential motor task “fist-edge-palm”, while the complex condition involved a coordinated motor-cognitive sequence (“bunny through ring”), requiring continuous switching between hand configurations and increased executive control.

Each task block lasted 1 min and was separated by 1 min rest intervals. The experimental sequence was repeated three times following an initial resting baseline recording.

Prior to each task block, participants were briefly shown a schematic instruction on the monitor. Task execution was then performed from memory without additional visual guidance. The complex task was designed to increase cognitive-motor integration demands, motor coordination complexity, and executive control requirements.

A detailed demonstration of the cognitive and motor-cognitive tasks is provided in Video S1 (cognitive and motor-cognitive tasks).

All participants were right-handed according to the demographic questionnaire. The motor task was performed with the non-dominant (left) hand, whereas the PPG sensor and skin-conductance electrodes were attached to the dominant (right) hand, which remained relaxed and did not participate in task execution.

### 2.3. Physiological Signal Acquisition

**Figure 2:**
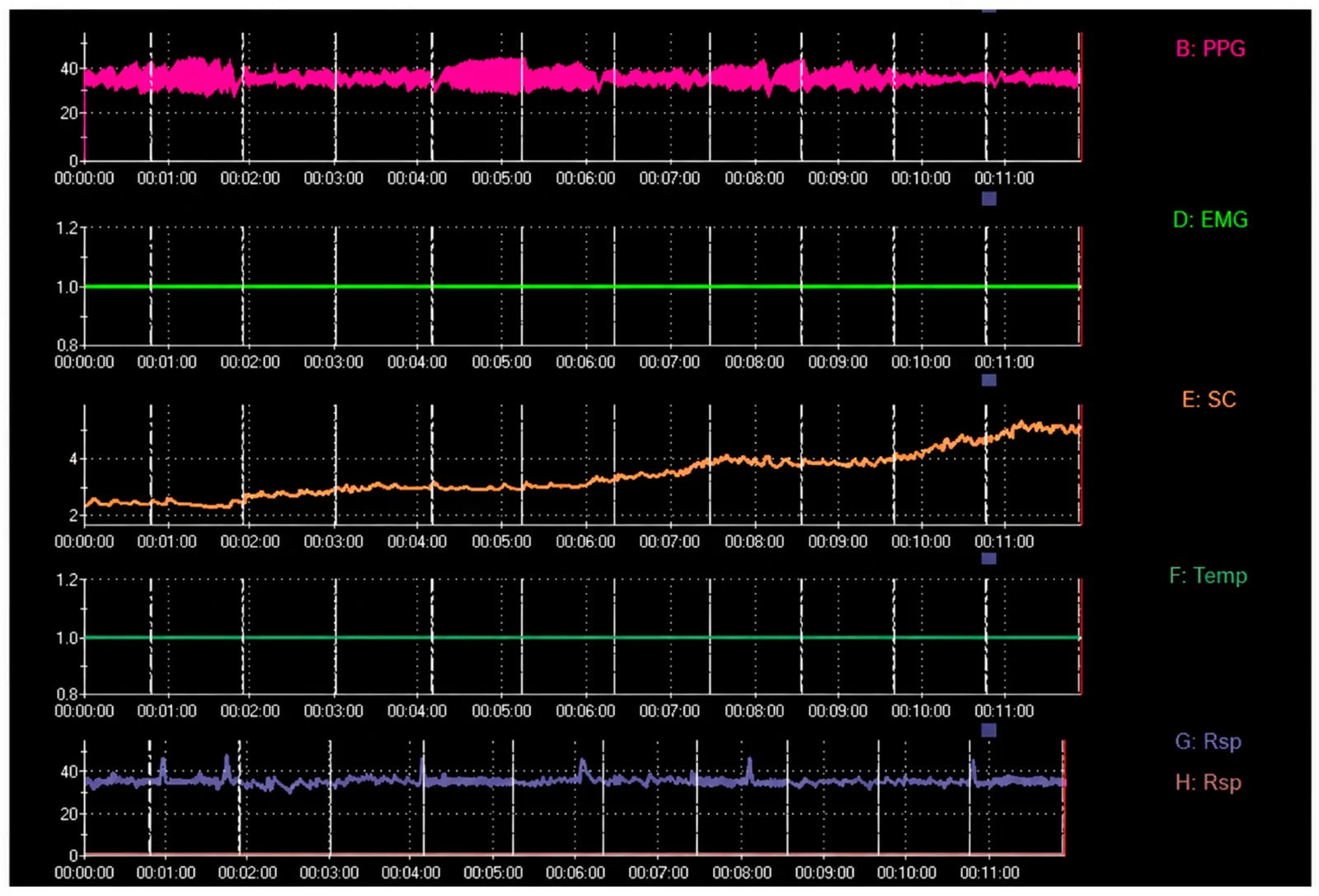
Representative screenshot of physiological signal acquisition using the Thought ProComp/FlexComp Infiniti system during the experimental session. Continuous recordings of photoplethysmography (PPG), skin conductance (SC), and respiration were obtained throughout the protocol. The white vertical lines represent manually inserted event markers used to delimit consecutive 1 min experimental stages. Note: EMG and temperature channels shown in the software interface were not used in the present study and are displayed only as part of the recording environment.

Physiological recordings were performed using a Thought Technology Ltd. (Montreal, QC, Canada) system, comprising a FlexComp Infiniti recording module and BioGraph Infiniti software (version 6.9; Thought Technology Ltd., Montreal West, QC, Canada). Data were transmitted wirelessly via Bluetooth Low Energy (BLE, 2.4 GHz) to a personal computer in real time, which minimized movement-related artifacts caused by cable tension (Figure 2).

The following physiological parameters were continuously recorded:

- Skin conductance (SC);
- Photoplethysmography (PPG);
- Respiration (abdominal breathing).

All physiological channels (PPG, SC and respiration) were acquired synchronously using the FlexComp Infiniti encoder at 2048 Hz.

Skin conductance was recorded using the Skin Conductance Flex/Pro sensor (SA9309M; Thought Technology Ltd., Montreal, QC, Canada) with Ag/AgCl finger electrodes attached to the distal phalanges of the second and fourth fingers. Skin conductance was measured using the exosomatic constant-voltage method (0.5–1.0 V), with a measurement range of 0–30 µS, in accordance with the manufacturer’s specifications. For each one-minute stage, the mean skin conductance level (SCL, µS) was calculated automatically and used for subsequent statistical and machine-learning analyses.

Photoplethysmography was recorded using the HR/BVP Flex/Pro photoplethysmography sensor (SA9308M; Thought Technology Ltd., Montreal, QC, Canada) attached to the thumb. The sensor provides the blood volume pulse (BVP) waveform, BVP amplitude, heart rate, and pulse-to-pulse (PP) intervals, from which heart rate variability (HRV) parameters were subsequently derived.

During the motor-cognitive protocol, both the SC and PPG sensors were attached to the hand that did not perform the movement task to minimize motion-related artifacts associated with repetitive hand movements.

Respiration was recorded using the Respiration Flex/Pro sensor (SA9311M; Thought Technology Ltd., Montreal, QC, Canada), a sensitive stretch-based girth sensor positioned around the abdomen. The sensor detects abdominal expansion and contraction and provides the respiration waveform, respiration amplitude, and respiration rate. The respiration signal represents a relative measure of abdominal expansion in accordance with the manufacturer’s specifications. For each one-minute stage, the mean respiration amplitude and mean respiration rate were calculated automatically and used for subsequent statistical and machine-learning analyses. Abdominal breathing was selected because diaphragmatic respiratory movements are closely associated with autonomic regulation and constitute a major component of slow deep breathing patterns that influence parasympathetic activity [27].

During recording, signal quality was visually monitored in real time. To minimize artifacts, participants were instructed to remain relatively still, avoid unnecessary hand and body movements, and maintain a calm, natural breathing rhythm outside the task-specific conditions. Recording segments containing pronounced movement-related or technical artifacts (e.g., sensor contact loss, abrupt non-physiological signal jumps, or temporary signal dropout) were excluded from further analysis.

After PPG acquisition, the signal was processed to analyze heart rate variability (HRV). It has been shown that spectral characteristics of HRV derived from pulse wave (PPG) are comparable to those calculated from electrocardiography [28,29]; therefore, PPG can be used as an alternative data source for HRV assessment.

From the raw PPG signal, a sequence of pulse-to-pulse (PP) intervals was extracted using the built-in functions of BioGraph Infiniti software. The resulting PP interval series was exported in a format compatible with Kubios HRV software, where it underwent preprocessing, including visual inspection, artifact detection, and exclusion of physiologically implausible intervals. Conventional heart rate variability (HRV) indices were subsequently calculated in Kubios HRV from the PPG-derived interval series using its standard processing pipeline. These indices included the mean RR interval (derived from the PPG-derived PP interval series), heart rate (HR), SDNN, RMSSD, Total Power, SD1, SD2, and related parameters.

Although standard HRV analysis commonly uses longer recordings, previous studies and current HRV recommendations indicate that ultra-short (1 min) recordings can provide reliable estimates of time-domain indices such as SDNN and RMSSD, while frequency-domain measures should be interpreted with greater caution. Total Power and SD2 were therefore included as relative descriptors of autonomic dynamics rather than as clinical HRV estimates [30].

Pulse peaks were detected using the built-in BioGraph Infiniti peak-detection algorithm. PP intervals were exported to Kubios HRV Standard (version 3.5.0) [11], where automatic medium-level artifact correction and cubic spline interpolation were applied.

The following physiological parameters were used for model building (Table 1).

### 2.4. Data Preprocessing and Feature Extraction

Physiological data preprocessing was performed using Python (version 3.12.13) in the Google Colab environment. PP interval preprocessing, including artifact correction and detrending, was performed using Kubios HRV Standard as described in Section 2.3.

Missing values, physiologically implausible observations, and artifacts were identified before analysis. Empty cells, textual representations of missing data, and zero values that were physiologically implausible for the corresponding variables were treated as missing values (NaN). Outliers were detected using a robust criterion based on the median absolute deviation (MAD; median ± 3.5 × 1.4826 × MAD) [31]. When MAD approached zero, the interquartile range (IQR) criterion was applied. Outliers were corrected using winsorization by replacing values outside the acceptable range with the nearest boundary value. Missing values were imputed using the median of the corresponding variable. Dynamic (Δ%) features were then calculated from the preprocessed dataset. Finally, all predictor variables were standardized using z-score normalization before machine learning model training.

Two groups of physiological features were extracted:

- Absolute physiological values;
- Dynamic physiological features.

Dynamic features were calculated as relative changes between consecutive experimental stages associated with increased task difficulty using the following formula:

Δ% = ((X_current_ − X_previous_)/X_previous_) × 100%

where X_previous_ corresponds to the value from the immediately preceding stage with lower load, and X_current_ corresponds to the value from the subsequent stage with higher load. Specifically, dynamic features were computed for the following transitions:

- Rest/baseline → simple task;
- Simple task → complex task.

Transitions involving a decrease in difficulty (e.g., complex task → rest) were not used for dynamic feature extraction in the classification of high-load stages.

Accordingly, the class labels for the dynamic-feature models represented these two transition types. The simple-transition class corresponded to task engagement following baseline/rest, whereas the complex-transition class corresponded to an increase in demand during ongoing task performance. Thus, transition type, preceding condition, and task-demand level were not experimentally independent in the dynamic-feature dataset. Dynamic-model performance was therefore interpreted as discrimination between these two standardized transition-related physiological responses rather than as context-independent classification of task complexity.

All recordings were visually inspected prior to feature extraction, and segments with obvious technical artifacts or temporary sensor-contact loss were excluded from further analysis.

### 2.5. Machine Learning Classification

Machine learning analysis was performed in Python (Google Colab) using the scikit-learn and statsmodels libraries. Three supervised classification algorithms representing complementary modeling approaches were evaluated: Logistic Regression (LR), Random Forest (RF), and Support Vector Machine (SVM). Logistic Regression served as an interpretable linear baseline classifier, whereas Random Forest and Support Vector Machine were selected because of their ability to capture nonlinear relationships frequently observed in physiological data while maintaining robust performance on datasets of moderate size [24,32]. Deep learning approaches were intentionally not considered because the primary objective of the present study was not to maximize predictive performance but to compare physiologically interpretable feature representations using transparent and reproducible machine learning methods. Unless otherwise specified, all classifiers were implemented using the default hyperparameters provided by the scikit-learn library to ensure fair comparison between algorithms and minimize model-specific optimization bias.

Hyperparameter optimization was intentionally not performed because the objective of the study was to compare feature representations rather than maximize predictive performance. Gradient boosting methods (e.g., XGBoost and LightGBM) were not included because the objective of the study was not algorithm benchmarking but comparison of physiologically interpretable feature representations using widely established machine-learning approaches.

Classification models were developed independently for (1) the cognitive protocol, (2) the motor-cognitive protocol, and (3) the combined dataset comprising both activity modalities. For each dataset, two alternative feature representations were evaluated. The first consisted of absolute physiological values (Abs) recorded at each experimental stage.

The second representation comprised dynamic physiological features (Δ%), calculated as the relative percentage change in each physiological parameter with respect to the immediately preceding experimental stage. In this dataset, class 0 represented the baseline/rest-to-simple transition and class 1 represented the simple-to-complex transition. Dynamic features were therefore intended to distinguish physiological responses associated with these two repeatedly reproduced transition types while reducing the influence of interindividual baseline variability.

Prior to model training, all predictor variables were standardized using z-score normalization. The dataset was randomly divided into training (80%) and testing (20%) subsets at the participant level, ensuring that physiological recordings from the same participant were never simultaneously included in both subsets and thereby preventing subject-related data leakage. A single participant-level split was used for the reported analysis; cross-validation was not performed. The same training procedure was applied to all evaluated classifiers to ensure an unbiased comparison of model performance.

Model performance was primarily evaluated using ROC-AUC because it is less sensitive to class imbalance and provides a threshold-independent estimate of discriminative performance. Classification accuracy is reported as a complementary performance measure.

To improve the physiological interpretability of the classification models, feature importance was estimated for the Random Forest classifier using the built-in Gini impurity-based importance measure. This analysis quantified the relative contribution of individual physiological variables to task-complexity classification and enabled identification of the physiological systems contributing most strongly to model performance. Particular attention was paid to physiologically interpretable cardiovascular and autonomic regulation features, including HRV, SC, PPG, and respiratory parameters, as these represented the primary physiological markers investigated in the present study.

Finally, classification performance obtained using models trained on absolute physiological values was compared with that of models trained on dynamic physiological features to compare stage-based discrimination using absolute physiological values with transition-based discrimination using dynamic physiological features within the fixed experimental sequence.

### 2.6. Assessment of Subjective Workload Using NASA-TLX

After completing each protocol, participants rated the subjective difficulty of the simple and complex task segments using the Raw NASA Task Load Index (NASA TLX). Ratings were obtained separately for the cognitive and motor-cognitive protocols. The scale comprised six dimensions (Mental, Physical, Temporal demand, Performance, Effort, Frustration), each rated on a 100-point scale. Performance was reverse-scored. No pairwise weighting was applied. Raw scores were summed to produce a global workload score per task segment per protocol per participant. These ratings were used to verify the effectiveness of the difficulty manipulation and to support interpretation of the physiological classification results. Subjective workload was assessed using a Russian-language adaptation of the NASA Task Load Index (NASA-TLX) developed and validated for Russian-speaking participants [33].

### 2.7. Objective Task-Performance Assessment

Objective task performance was assessed retrospectively from video recordings by two independent experts with expertise in neurophysiology. The experts were not involved in conducting the experiment and were not present during data collection. Each recording was evaluated independently using a standardized scoring form and predefined assessment criteria. In cases of disagreement, the experts reviewed the recording jointly and reached a consensus.

Performance was evaluated separately for each one-minute task stage. For the Reading protocol, reading speed was calculated as the number of words read per minute. For the Movement protocol, execution speed was calculated as the number of completed movement elements per minute. A movement element was defined as one hand configuration within the predefined sequence. Thus, one complete “fist–edge–palm” sequence comprised three elements, whereas one complete “bunny-through-ring” sequence comprised thirteen elements. Element-based scoring was used because the two exercises differed substantially in sequence length and completion time, making the number of complete sequences per minute unsuitable for direct comparison.

Incorrect and incomplete responses were coded separately by the experts using predefined criteria. Because the present study focused on task-execution speed as an objective measure supporting the experimental manipulation, error-related outcomes were not included in the current analysis and will be examined separately.

## 3. Results

### 3.1. Classification Results for the Cognitive (Reading) Protocol

All classification metrics reported in Section 3.1, Section 3.2 and Section 3.3 were calculated on the held-out participant-level test set comprising 20% of the participants.

For the reading protocol, classification of cognitive task difficulty using absolute physiological values showed low performance regardless of the model used (ROC-AUC = 0.412–0.562). This indicates that the absolute level of physiological activation does not accurately reflect the differences between simple and complex stages of cognitive activity.

**Figure 3:**
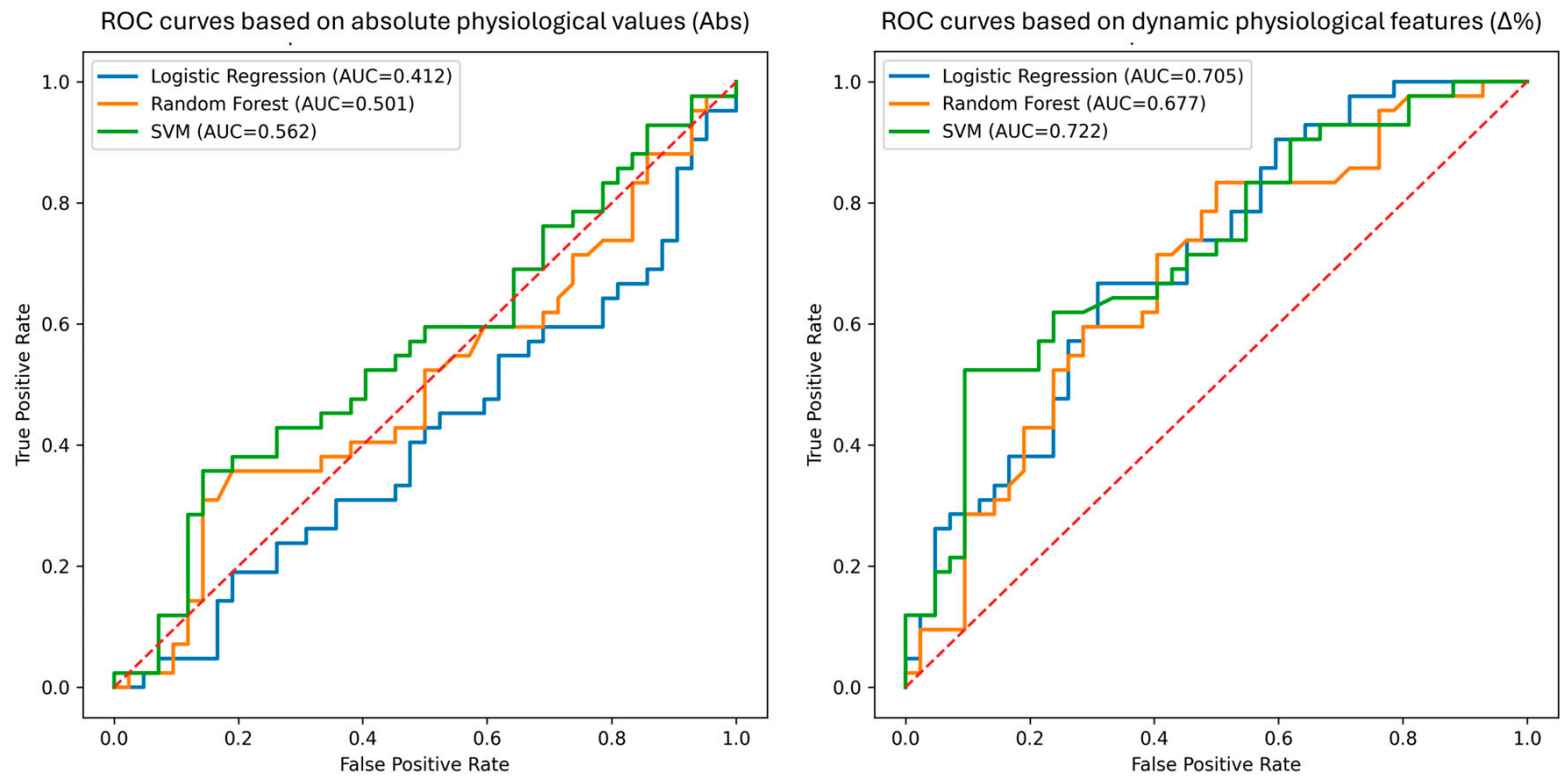
ROC curves for classification models of cognitive task complexity (Reading protocol). The left shows discrimination between physiological states recorded during simple and complex task stages using absolute values. The right shows discrimination between baseline/rest-to-simple and simple-to-complex transitions using dynamic physiological features (Δ%). Note: Results are shown for Logistic Regression, Random Forest, and SVM.

In contrast, dynamic physiological features provided substantially better discrimination between baseline/rest-to-simple and simple-to-complex transitions (Δ, % relative to the previous stage). The best result was achieved by the Support Vector Machine (SVM) model (Accuracy = 0.655; ROC-AUC = 0.722). Logistic Regression also demonstrated acceptable classification quality (ROC-AUC = 0.705), while Random Forest showed slightly lower values (ROC-AUC = 0.677) (Figure 3).

While the ROC curves illustrate the overall discriminative ability of each model, the exact classification performance metrics are presented in Table 2, which also confirms the consistent improvement achieved with Δ-based features.

**Figure 4:**
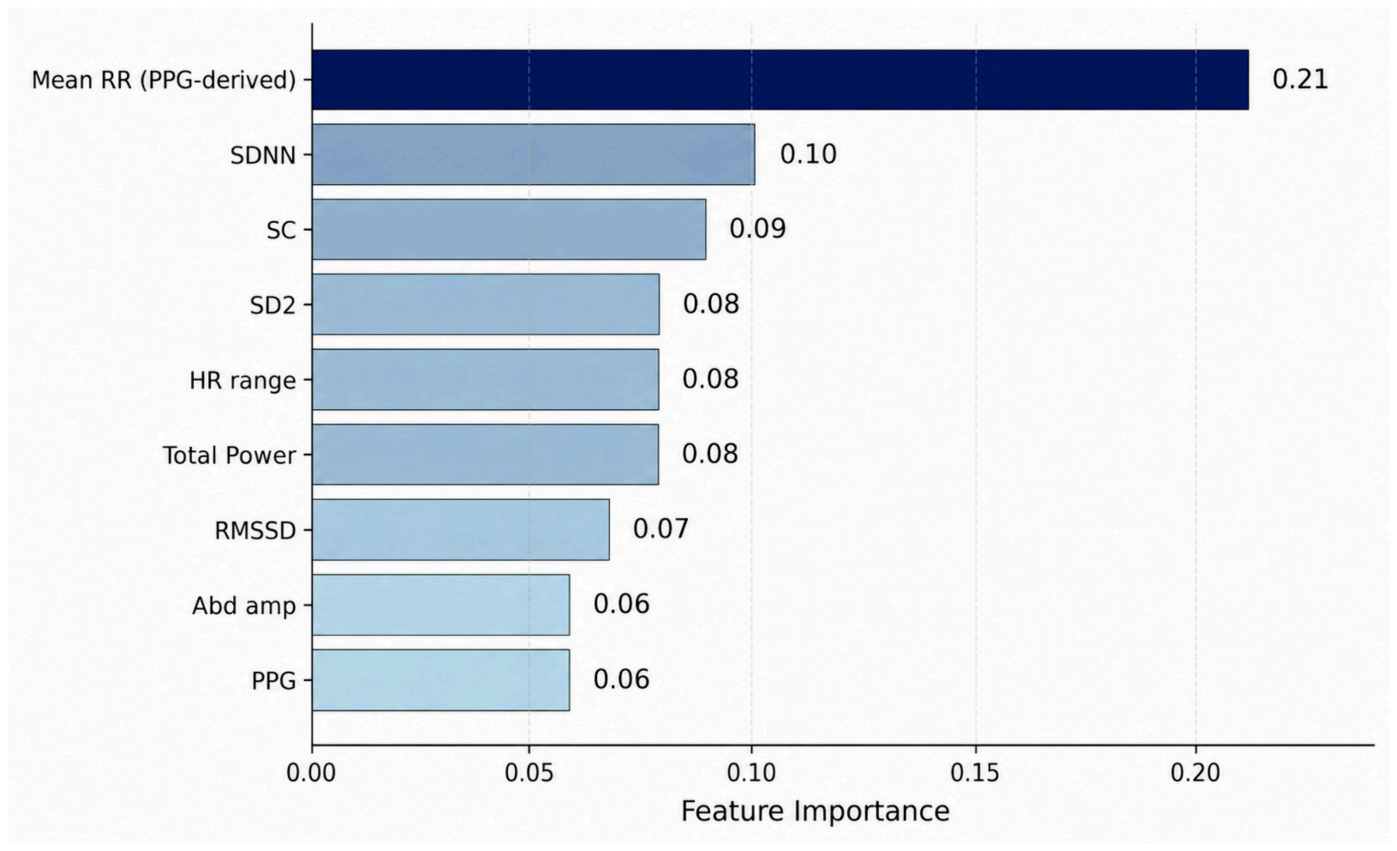
Most informative Δ-based features of physiological regulation in the Random Forest model for discrimination between baseline/rest-to-simple and simple-to-complex transitions in the Reading protocol. Note: Importance reflects the relative contribution of physiological features to the model’s classification performance.

Feature-importance analysis revealed that the most informative Δ-based feature for classification of the reading-task stages was the mean RR interval derived from the PPG-derived PP interval series (importance = 0.213), followed by SDNN (0.103), SC (0.091), Total Power (0.083), HR range (0.081), SD2 (0.079), RMSSD (0.071), PPG (0.060), and abdominal amplitude (0.058) (Figure 4).

### 3.2. Classification Results for the Motor-Cognitive (Movement) Protocol

**Figure 5:**
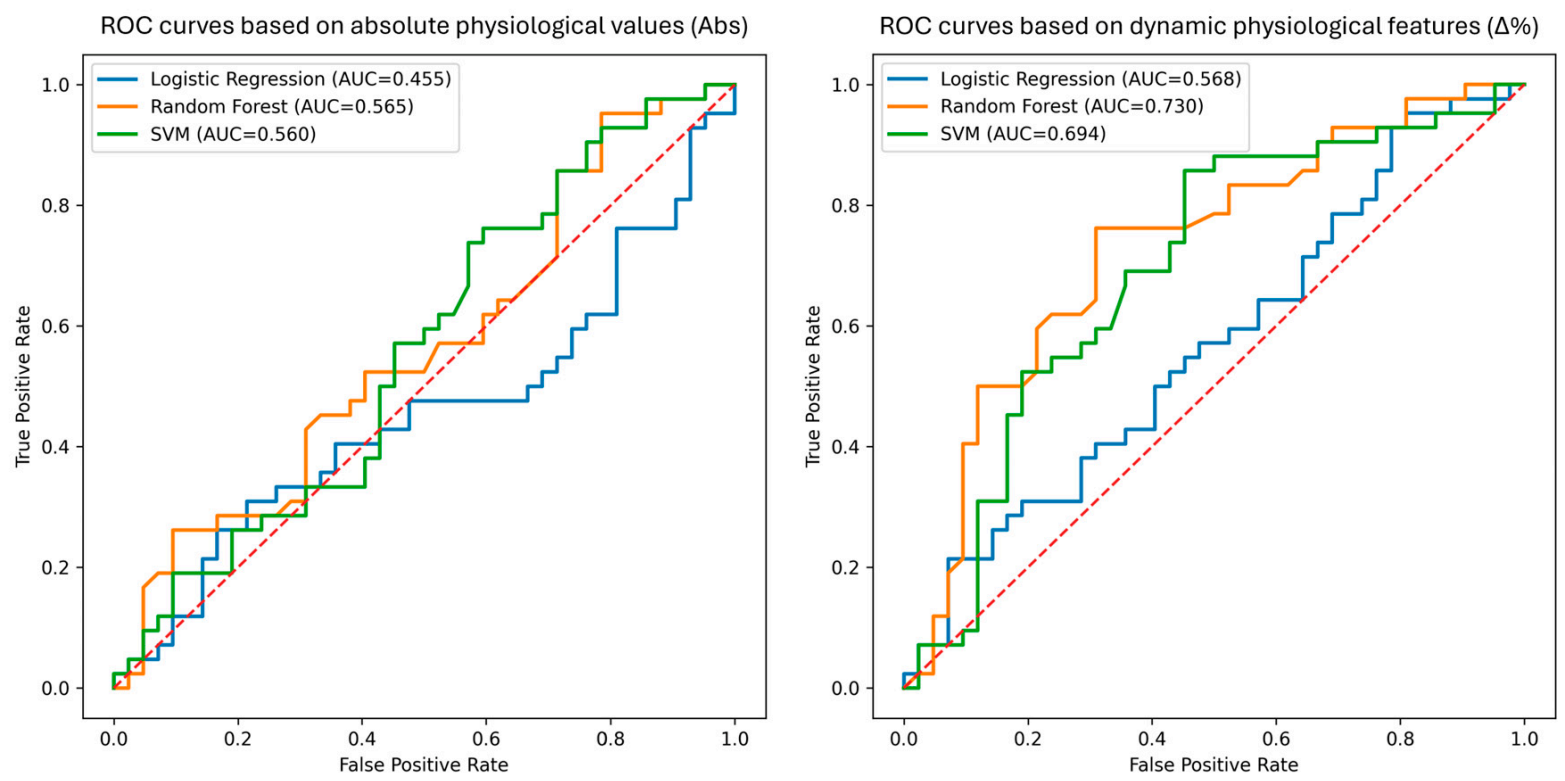
ROC curves for classification models of motor-cognitive task complexity (Movement protocol). The left shows discrimination between physiological states recorded during simple and complex task stages using absolute values. The right shows discrimination between baseline/rest-to-simple and simple-to-complex transitions using dynamic physiological features (Δ%). Note: Results are shown for Logistic Regression, Random Forest, and SVM.

For the motor-cognitive protocol, classification based on absolute physiological values showed low performance regardless of the model used (ROC-AUC = 0.455–0.565). This indicates that the absolute level of physiological activation does not accurately reflect the differences between simple and complex motor tasks (Figure 5).

In contrast, using dynamic physiological features (Δ, % relative to the previous stage) substantially improved discrimination between baseline/rest-to-simple and simple-to-complex transitions. The best result was achieved by the Random Forest model (Accuracy = 0.726; ROC-AUC = 0.730). The Support Vector Machine (SVM) also provided high classification performance (Accuracy = 0.667; ROC-AUC = 0.694), outperforming the linear Logistic Regression model (Table 3).

**Figure 6:**
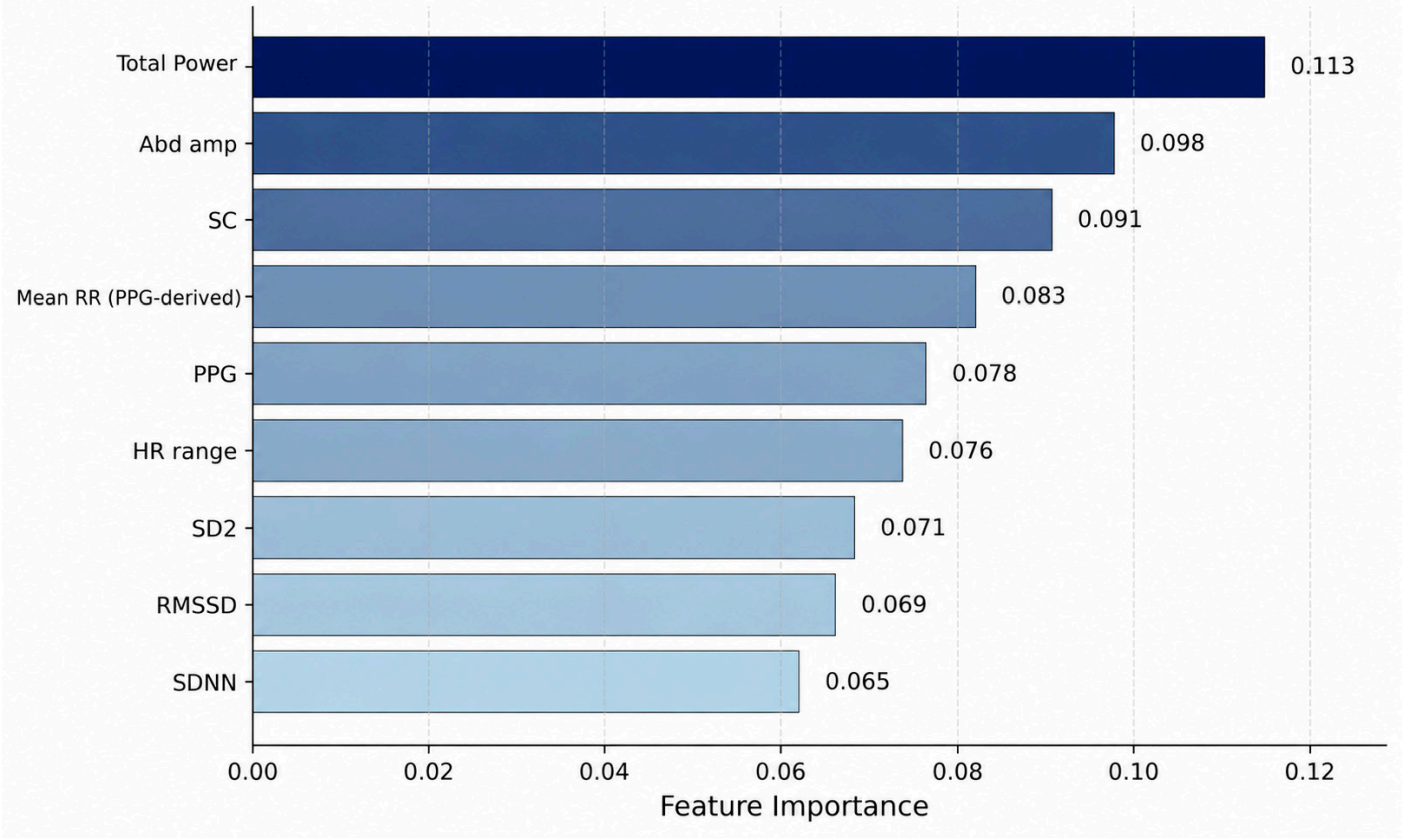
Most informative Δ-based features of physiological regulation in the Random Forest model for discrimination between baseline/rest-to-simple and simple-to-complex transitions in the Movement protocol. Note: Importance reflects the relative contribution of physiological features to the model’s classification performance.

Figure 6 presents the feature importance of the Δ-based physiological variables in the Random Forest model for the movement protocol. Total Power, abdominal respiration amplitude, SC, mean PPG-derived RR interval, and PPG amplitude contributed most to the classification performance, whereas HR range, SD2, RMSSD, and SDNN showed moderate but consistent importance.

### 3.3. Classification Results for the Combined Dataset (Cognitive and Motor-Cognitive Protocols)

**Figure 7:**
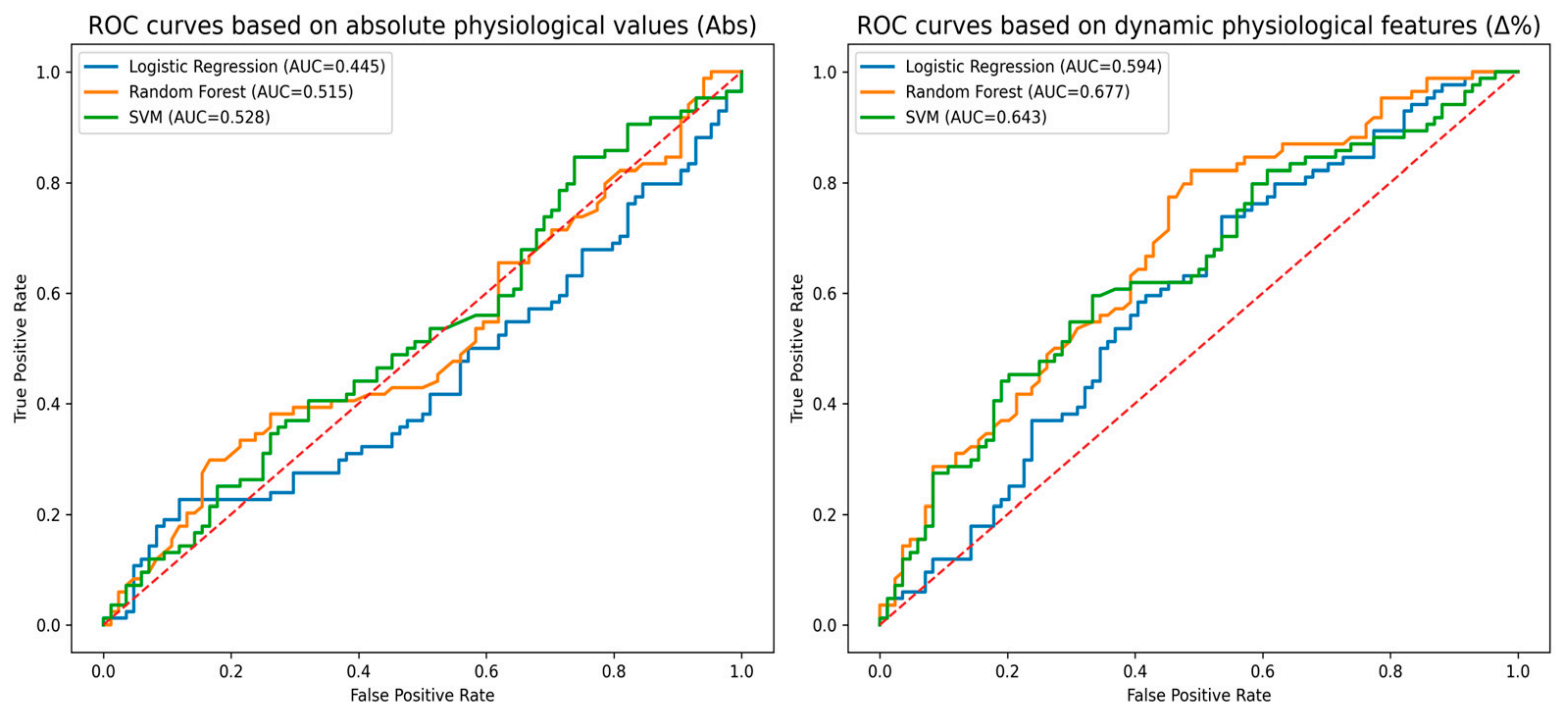
ROC curves for classification models of task complexity based on a combined (reading and movement) dataset. The left shows discrimination between physiological states recorded during simple and complex task stages using absolute values. The right shows discrimination between baseline/rest-to-simple and simple-to-complex transitions using dynamic physiological features (Δ%). Note: Results are shown for Logistic Regression, Random Forest, and SVM.

After combining the data obtained from the two protocols, the models trained on dynamic physiological features (Δ-values) showed better discrimination between the two standardized transition types than models based on absolute values showed between the corresponding simple and complex task stages. Random Forest achieved the highest ROC-AUC (0.677), whereas SVM achieved the highest classification accuracy (0.619). (Figure 7).

The following are the exact indicators of ML classification efficiency for the combined dataset (Table 4).

**Figure 8:**
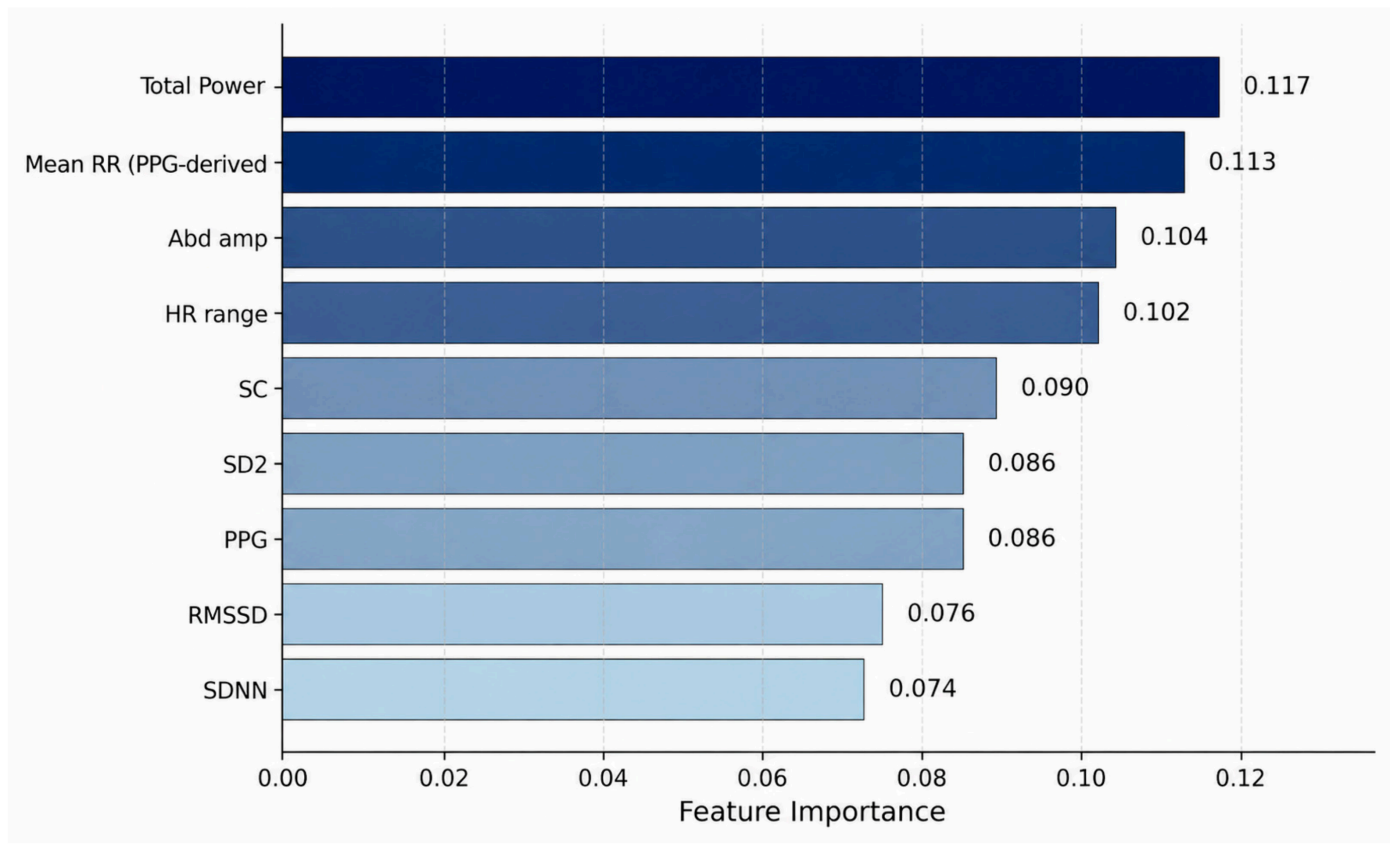
Most informative Δ-based features of physiological regulation in the Random Forest model for discrimination between baseline/rest-to-simple and simple-to-complex transitions in the combined (reading and movement) dataset. Note: Importance reflects the relative contribution of each feature to transition classification.

Analysis of feature importance showed that total power, the mean RR interval, HR range, abdominal respiration amplitude and SD2 were among the most influential predictors. The predominance of dynamic characteristics associated with HRV suggests that the autonomous regulatory adjustments that occur during the transition from one task to another contain information that contributes to discrimination between task engagement following recovery and a subsequent increase in task demand during ongoing activity. (Figure 8).

### 3.4. Subjective Workload Assessment (NASA TLX)

**Figure 9:**
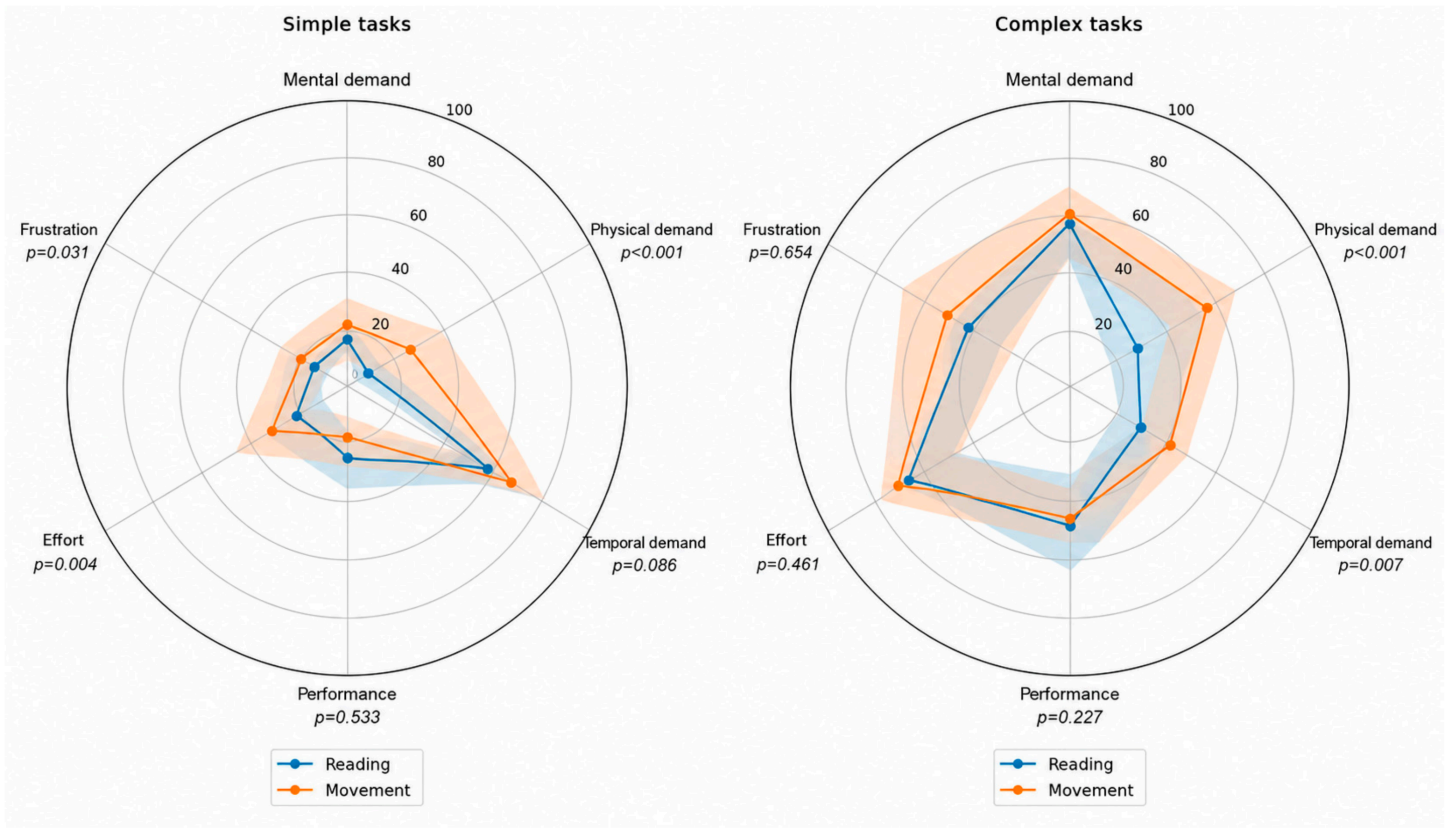
NASA-TLX ratings for simple (left) and complex (right) tasks in the reading and movement protocols. Lines represent median values, and shaded areas indicate the interquartile range (Q1–Q3). Significant between-protocol differences are indicated by *p*-values.

**Figure 10:**
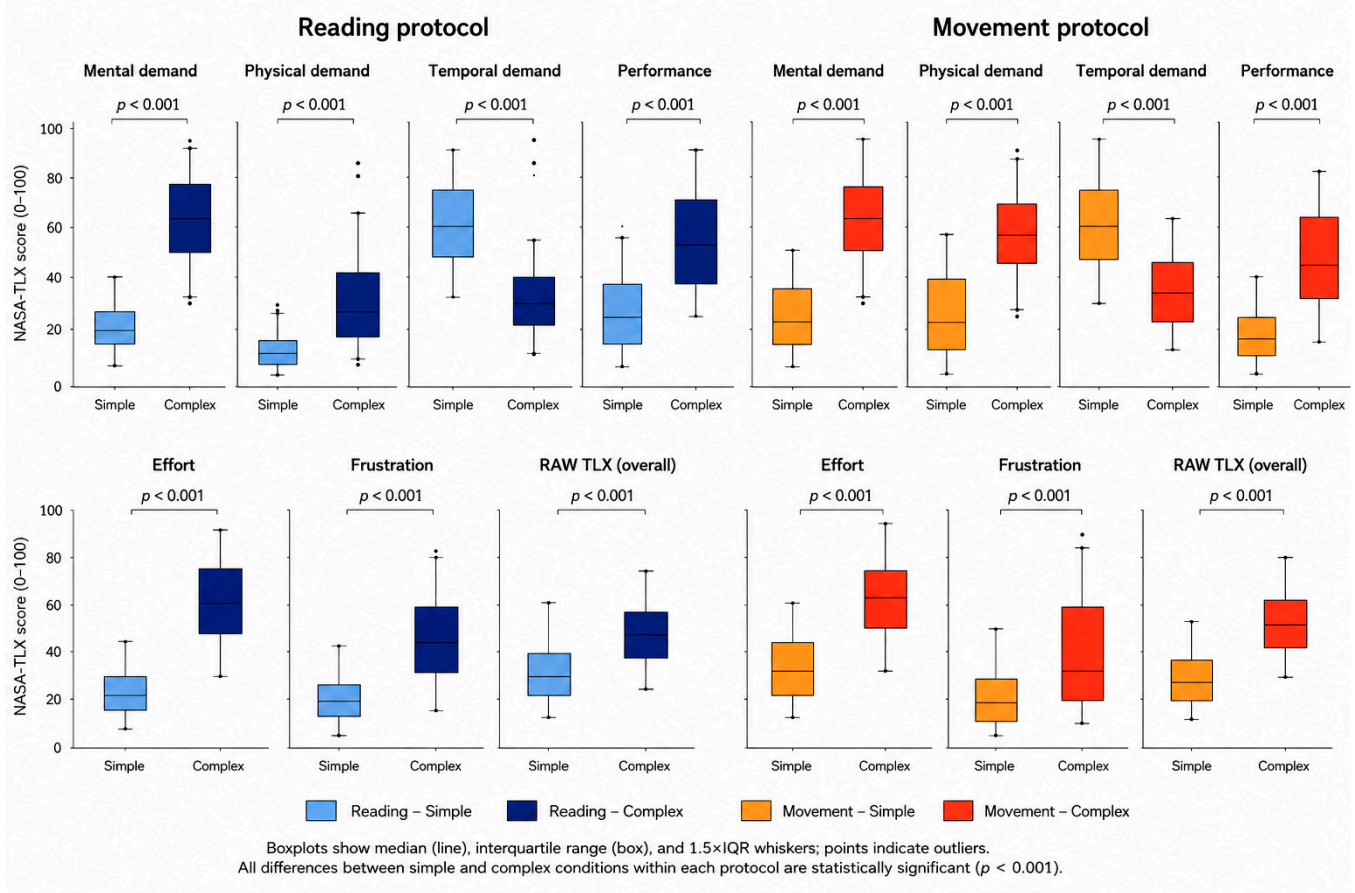
Comparison of subjective workload assessed using the NASA Task Load Index (NASA-TLX) between simple and complex task conditions within the Reading and Movement protocols. Boxplots show median values, interquartile ranges (Q1–Q3), 1.5 × IQR whiskers, and outliers. Within each protocol, complex tasks differed significantly from simple tasks across all NASA-TLX dimensions and the overall RAW TLX score (*p* < 0.001).

To verify the effectiveness of the experimental manipulation of task complexity and to support the interpretation of physiological classification results, participants completed the Raw NASA Task Load Index (NASA-TLX) after each protocol. The questionnaire confirmed that the complex task segments were perceived as substantially more demanding than the simple segments in both protocols (Figure 9 and Figure 10).

For simple tasks, the movement protocol was associated with significantly higher physical demand, effort, and frustration compared with the reading protocol, whereas no significant differences were observed for mental demand, temporal demand, or perceived performance. These findings indicate that even under low-complexity conditions, the motor-cognitive task imposes a greater physical and operational workload.

For complex tasks, physical demand remained significantly higher during the movement protocol, and temporal demand was also elevated relative to the reading protocol. In contrast, no significant between-protocol differences were observed for mental demand, effort, performance, or frustration. Notably, both protocols elicited similarly high ratings of mental demand and effort during complex task performance.

Overall, the NASA-TLX results support the validity of the task complexity manipulation and demonstrate that the complex task conditions were consistently associated with greater subjective workload.

### 3.5. Objective Task Performance

**Figure 11:**
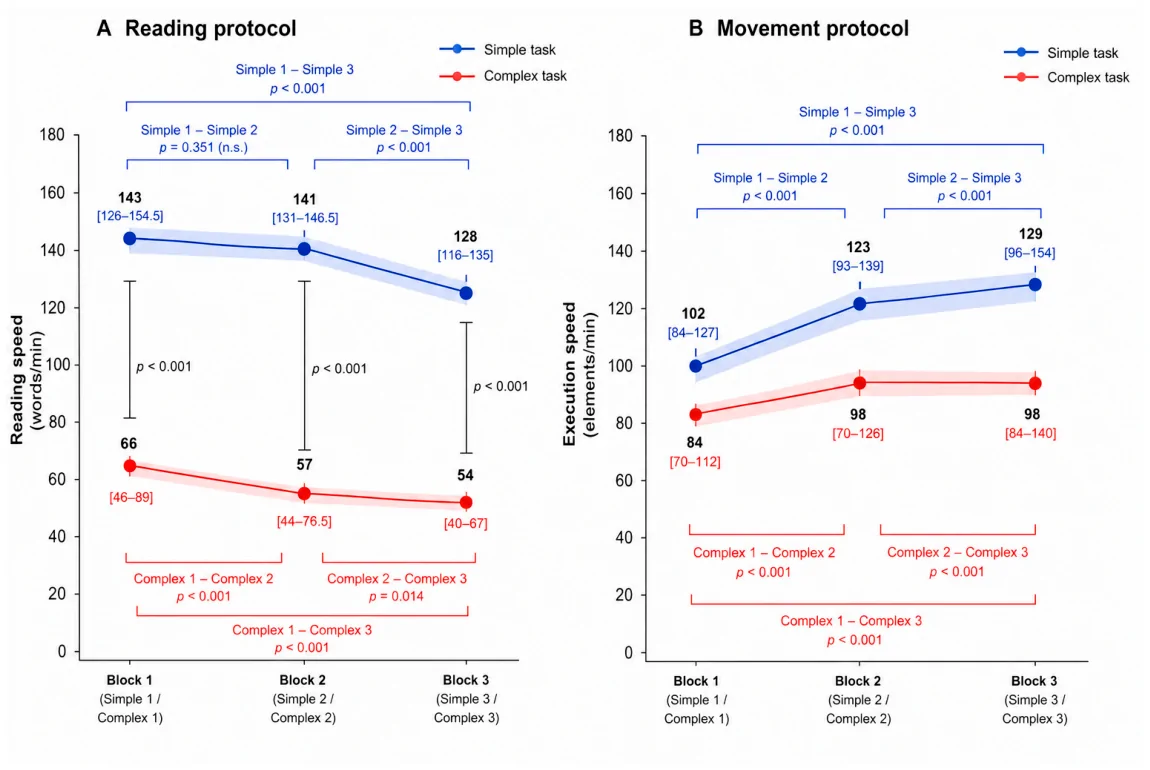
Objective task performance in the Reading (**A**) and Movement (**B**) protocols. Median values are connected by lines, and shaded areas represent the interquartile range (Q1–Q3). Horizontal brackets indicate within-condition comparisons across repeated task blocks, whereas vertical brackets indicate comparisons between the corresponding simple and complex task stages. Statistical significance was assessed using the Wilcoxon signed-rank test.

To verify that the experimental manipulation of task complexity produced measurable changes in behavioral performance, objective task-execution metrics were analyzed for both protocols (Figure 11).

In the Reading protocol, reading speed was significantly lower during all complex task stages than during the corresponding simple task stages (all Wilcoxon tests, *p* < 0.001). Median reading speed decreased from 143 [126–154.5], 141 [131–146.5], and 128 [116–135] words/min during the three simple stages to 66 [46–89], 57 [44–76.5], and 54 [40–67] words/min during the corresponding complex stages, confirming the effectiveness of the complexity manipulation.

In the Movement protocol, execution speed also differed between task conditions. Although complex motor sequences were consistently performed more slowly than simple sequences, both task conditions demonstrated progressive increases in execution speed across repeated trials, reflecting practice-related adaptation. Median execution speed increased from 102 [84–127] to 129 [96–154] elements/min across the simple stages and from 84 [70–112] to 98 [84–140] elements/min across the complex stages. Improvements between repeated stages were statistically significant for both simple and complex conditions (all *p* < 0.001).

## 4. Discussion

The present study evaluated the feasibility of ML-based classification of task complexity using physiological signals recorded during cognitive and motor-cognitive activities.

Both subjective (NASA-TLX) and objective (task-execution speed) measures confirmed the effectiveness of the experimental manipulation, supporting the interpretation that the observed physiological differences primarily reflected changes in task demands rather than random fluctuations in performance.

The main finding was that models trained on dynamic physiological features consistently outperformed those based on absolute physiological values in the reading and movement protocols and in the combined dataset.

However, the dynamic classes represented baseline/rest-to-simple and simple-to-complex transitions within a fixed experimental sequence. Their superior performance should therefore be interpreted as improved discrimination of these standardized transition-related responses rather than as evidence of context-independent task-complexity classification.

The study extends previous workload research by demonstrating that transition-based features provide consistent advantages across two distinct activity modalities. The partially similar feature patterns observed in the cognitive and motor-cognitive protocols suggest that comparable autonomic systems contribute to responses during standardized increases in task demand. Whether these patterns generalize to other transition contexts or randomized task sequences remains to be established.

One possible explanation is that absolute physiological values are highly susceptible to interindividual variability. Baseline autonomic activity varies with age, sex, fitness, lifestyle, and other factors, resulting in substantial between-subject variability in HRV and related indices [10]. Consequently, identical task demands may elicit different absolute responses across participants. By contrast, dynamic characteristics describe relative changes during transitions between task conditions and may therefore directly reflect task-related adaptation. This interpretation is consistent with previous research emphasizing workload transitions and adaptive physiological responses [34]. The findings support the view that autonomic adaptation should be interpreted as a dynamic process rather than as a sequence of independent states. Similar observations have been reported in real-world workload-monitoring studies, where individual calibration and change patterns were more informative than absolute physiological levels alone [25].

Feature-importance analysis provided additional insight into the physiological variables contributing to classification. In the combined model, the highest-ranked predictors included the PPG-derived mean RR interval, Total Power, abdominal respiration amplitude, HR range, skin conductance, and SD2. Their importance was relatively evenly distributed across physiological domains, with no single variable showing overwhelming dominance.

This pattern suggests that increasing task demands are associated with a distributed autonomic response involving several physiological systems. HRV indicators, including the mean RR intervals, Total Power, HR range, SD2, RMSSD, and SDNN, were consistently represented among the most informative predictors, indicating an important contribution of cardiac autonomic regulation. Respiratory amplitude and skin conductance also contributed substantially, particularly in the combined and motor-cognitive datasets, suggesting that respiratory and sympathetic electrodermal responses provide complementary information.

The balanced distribution of feature importance across cardiovascular, electrodermal, respiratory, and peripheral vascular variables argues against a single dominant marker of task complexity. Instead, adaptation appears to emerge from coordinated interactions among multiple autonomic subsystems. This supports the development of multimodal monitoring systems that integrate complementary biosignals rather than relying on a single measure. Previous studies have similarly shown that workload-related responses arise from interactions among several physiological systems [8,16], and that multimodal approaches improve cognitive-load assessment by capturing different aspects of adaptation to changing task requirements [26].

Taken together, the results indicate that task complexity is represented by a multimodal adaptation pattern involving coordinated cardiovascular, respiratory, electrodermal, and peripheral vascular adjustments.

The recurrence of similar feature patterns across the two protocols suggests partial overlap in the autonomic systems contributing to classification. However, because cross-protocol transfer was not evaluated, these findings should not be interpreted as evidence that a model trained on one activity modality would generalize to another.

The superior performance of Random Forest and Support Vector Machine compared with Logistic Regression suggests that the relationship between autonomic responses and the standardized task-stage or transition labels is inherently nonlinear. Similar findings have been reported in previous workload studies, in which nonlinear ML methods outperformed linear approaches when classifying cognitive states and workload levels [19,20]. Logistic Regression provided an interpretable linear baseline, whereas Random Forest and Support Vector Machine were better suited to model multidimensional interactions among autonomic variables.

The best-performing classifier differed between activity modalities. Support Vector Machine achieved the highest performance in the cognitive protocol, whereas Random Forest performed best in the motor-cognitive protocol. Although these differences should be interpreted cautiously, they suggest that optimal model performance may depend on the structure of the underlying responses. Cognitive tasks may have generated relatively compact response patterns that favored Support Vector Machine classification, whereas the greater interindividual variability and heterogeneity associated with motor-cognitive activity may have been better captured by the ensemble-based Random Forest classifier. This interpretation is consistent with evidence that different ML algorithms capture complementary aspects of physiological regulation depending on task characteristics and data structure [18,20].

An important practical advantage is that the reported performance was achieved using physiologically interpretable features and relatively simple ML models. The results therefore support the feasibility of monitoring systems capable of detecting responses resembling the standardized task-demand transitions examined here. Nevertheless, validation in randomized, counterbalanced, and real-world task sequences is required before the models can be used for context-independent detection of task complexity.

Many of the highest-ranked predictors can be obtained using wearable cardiovascular monitoring systems without complex neurophysiological instrumentation. This enhances the translational potential of the approach for occupational, educational, and rehabilitation settings. The prominent contribution of the PPG-derived mean RR interval and other HRV-related features is particularly encouraging because cardiovascular signals can be acquired continuously using widely available wearable sensors. Such systems may support adaptive training, operator monitoring, rehabilitation technologies, and other applications requiring objective assessment of functional state [3].

The reported performance estimates were obtained from a single held-out participant-level test set. Future studies should employ repeated validation procedures to estimate confidence intervals and quantify model variability.

Several limitations should be acknowledged. The sample consisted of healthy young adults, which may limit generalizability to other populations. The experimental tasks were laboratory models of cognitive and motor-cognitive activity and may not reproduce the variability of real operational environments. Real-world settings involve greater variation in task context, environmental conditions, concurrent demands, and individual behavioral strategies, all of which may affect model performance. Furthermore, although classification performance was consistently above chance level, it remained moderate. Larger datasets, additional modalities, and advanced temporal models may improve performance.

A major design limitation is that task order was fixed in both protocols. Dynamic-feature class labels were therefore intrinsically associated with both transition type and preceding condition: baseline/rest-to-simple for one class and simple-to-complex for the other. Consequently, the models may have captured effects related to task engagement, preceding autonomic state, carryover, learning, fatigue, or protocol position in addition to changes in task demand.

The fixed sequence was selected deliberately to provide three repeated and directly comparable observations of each predefined transition type. Randomizing individual stages would have generated additional transition combinations and reduced the number of observations available for each transition. Obtaining an equivalent number of observations in a fully counterbalanced design would have required a substantially longer protocol, potentially increasing fatigue and time-on-task effects. Nevertheless, the present findings cannot establish context-independent classification of task complexity. Future studies should evaluate simple and complex tasks after the same preceding condition and use randomized or counterbalanced sequences designed to separate task difficulty from transition context.

Cross-protocol transferability was not evaluated. Separate models were trained and tested within each protocol, whereas the combined model included data from both protocols during training and testing. Therefore, similarities in feature importance and model performance indicate overlapping physiological contributions but do not demonstrate that a classifier trained on one activity modality can generalize to another.

Only classical ML algorithms were evaluated. These models were selected because of their interpretability and suitability for moderate-sized datasets; however, future studies should determine whether temporal deep-learning architectures provide additional benefits without compromising physiological interpretability.

Quantitative condition-specific signal-quality indices were not retained during preprocessing; therefore, artifact rates could not be retrospectively compared between simple and complex motor conditions. Future studies should incorporate automated signal-quality metrics and condition-specific artifact reporting.

Feature importance was estimated using the impurity-based measure implemented in Random Forest. Although widely used, this method may be influenced by predictor characteristics and does not provide model-independent estimates. Future studies should evaluate permutation-based or SHAP-based explanations within repeated validation frameworks.

Further research should directly assess cross-protocol transfer by training models on one activity type and testing them on another. Continuous sliding-window approaches should also be investigated for real-time detection of transitions between activity states. Finally, the robustness of transition-based features should be evaluated in more ecologically valid operational scenarios and across task domains.

## 5. Conclusions

This study demonstrated that ML models can discriminate physiological states recorded during simple and complex task stages and physiological responses associated with two repeatedly reproduced task-demand transitions in cognitive and motor-cognitive protocols. Dynamic features provided superior classification performance for distinguishing baseline/rest-to-simple from simple-to-complex transitions than absolute physiological values provided for distinguishing the corresponding task stages.

The transition-based models relied on a distributed pattern involving cardiovascular, respiratory, electrodermal, and peripheral vascular features. Similar feature patterns across the Reading and Movement protocols suggest that partially shared autonomic components contribute to physiological responses during standardized increases in task demand.

However, because task order was fixed and transition type was directly linked to the class label, the dynamic models should not be interpreted as context-independent classifiers of task complexity. The results are limited to physiological responses observed during baseline/rest-to-simple and simple-to-complex transitions within the standardized sequence used in this study. Randomized and counterbalanced experiments are required to determine whether these physiological patterns generalize across preceding conditions, task orders, and real-world activity contexts.

## Figures and Tables

**Table 1:** Description of the physiological parameters used.

Parameter	Unit	Description
HR range	bpm	Difference between maximum and minimum heart rate within a 1 min recording period (HR max–HR min)
Mean RR interval (PPG-derived)	ms	Mean RR interval was calculated in Kubios HRV from PPG-derived pulse-to-pulse (PP) intervals.
SDNN	ms	Standard deviation of RR intervals
RMSSD	ms	Root means square of successive differences between adjacent RR intervals
Total power	ms^2^	Total power of heart rate variability
SD2	ms	Poincaré plot standard deviation along the line of identity
Abdominal amplitude (Abd_amp.)	a.u.	Amplitude of abdominal breathing
SC	μS	Skin conductance
PPG amplitude	a.u.	Photoplethysmography

**Table 2:** Classification performance for the cognitive (reading) protocol.

Dataset	Model	Accuracy	ROC-AUC
Task-stage absolute values	Logistic Regression	0.464	0.412
	Random Forest	0.524	0.501
	SVM	0.500	0.562
Transition-based Δ-based features	Logistic Regression	0.667	0.705
	Random Forest	0.619	0.677
	SVM	0.655	0.722

**Table 3:** Classification performance for the motor-cognitive (Movement) protocol.

Dataset	Model	Accuracy	ROC-AUC
Task-stage absolute values	Logistic Regression	0.488	0.455
	Random Forest	0.512	0.565
	SVM	0.464	0.560
Transition-based Δ-based features	Logistic Regression	0.536	0.568
	Random Forest	0.726	0.730
	SVM	0.667	0.694

**Table 4:** Classification performance for the combined (reading and movement protocols) dataset.

Dataset	Model	Accuracy	ROC-AUC
Task-stage absolute values	Logistic Regression	0.452	0.445
	Random Forest	0.470	0.515
	SVM	0.494	0.528
Transition-based Δ-based features	Logistic Regression	0.577	0.594
	Random Forest	0.595	0.677
	SVM	0.619	0.643

## Data Availability

The data presented in this study are available upon request from the corresponding author.

## Supplementary Materials

The following supporting information can be downloaded at https://www.mdpi.com/article/10.3390/bioengineering13080898/s1: Video S1: Cognitive and motor-cognitive tasks.

## Abbreviations

The following abbreviations are used in this manuscript:

HRV	Heart Rate Variability
PPG	Photoplethysmography
SVM	Support Vector Machine
SC	Skin Conductance
HR	Heart Rate
